# Continuous identification of the tea shoot tip and accurate positioning of picking points for a harvesting from standard plantations

**DOI:** 10.3389/fpls.2023.1211279

**Published:** 2023-10-11

**Authors:** Kun Luo, Xuechen Zhang, Chengmao Cao, Zhengmin Wu, Kuan Qin, Chuan Wang, Weiqing Li, Le Chen, Wei Chen

**Affiliations:** ^1^ College of Mechanical Engineering, Tongling University, Tongling, China; ^2^ Key Laboratory of Construction Hydraulic Robots of Anhui Higher Education Institutes, Tongling University, Tongling, China; ^3^ Advanced Copper-based Material Industry Generic Technology Research Center of Anhui Province, Tongling University, Tongling, China; ^4^ School of Engineering, Anhui Agricultural University, Hefei, China; ^5^ State Key Laboratory of Tea Plant Biology and Utilization, Anhui Agricultural University, Hefei, China; ^6^ Institute of Agricultural Engineering, Anhui Academy of Agricultural Sciences, Hefei, China

**Keywords:** tea shoots, continuous picking, two-dimensional imaging, low-damage picker, You Only Look Once

## Abstract

To address the current problems of large positioning error, low picking efficiency, and high cost of tea shoot picking, a continuous and precise harvesting scheme for tea shoots based on a two-dimensional (2D) perspective is designed in this study. A high-speed harvesting method for tea shoots in a standardized tea plantation assembly line type was proposed. First, a 2D view recognition model of tea shoot tips in a multi-disturbance environment was constructed, and accurate picking point coordinates were determined by combining a skeleton algorithm and curve growth. To avoid the losses of recognition accuracy caused by the mistaken clamping of blades and vibrations during harvester operations, accurate control of the harvester was realized by combining path planning and the S-curve speed control function. The recognition accuracy for the verification set of the recognition model was 99.9%, and the mean average precision (0.5:0.95) value was 0.97. The test results show that the error between the actual picking point position and the position determined by the model was within ± 3 mm, and the picking success rate was 83.6%. Therefore, we can realize fast and accurate picking of tea shoots and lay the foundation for continuous tea picking in the future by simplifying the identification and picking process.

## 1 Introduction

China’s tea production ranks first globally, and China is a big consumer of tea products. Hence, tea is an economically important crop for the country. Currently, the picking of tea shoots is primarily performed by hand, resulting in low picking efficiencies. In particular, 60% of the processing cost of tea is expended on fresh leaf acquisition. In addition, short picking times and harsh growing environments for tea leaves can result in personnel injuries during the manual picking of tea leaves, inconsistent tea leaf quality, untimely harvesting of large quantities of fresh leaves, and low picking yields, thereby increasing the prices for premium teas. Moreover, China’s countryside populations are aging and losing young workers, dramatically decreasing their labor forces. Thus, the shortage of labor and growing environmental constraints in the development of premium tea industry prompt the urgent need for a tea leaves picking equipment that can replace manual labor.

In recent years, scholars have explored systems used to harvest premium tea shoots. Xu L. et al. (2022) designed a picking box with clips cut on both sides. The Graham algorithm enabled the picking box to frame as many tea shoots as possible. Subsequently, tea leaves were clamped and cut, and the shoots were collected using negative-pressure airflows. Zhe (2020) studied the microstructures and cutting characteristics of tea stalks and the morphological structures of cricket epiglottal tangential leaves. Consequently, a bionic cutter was designed, and finite element analyses and cutting performance tests were conducted to optimize it. Zhu et al. (2021) designed a plucking device based on negative-pressure guidance for premium tea. The tender tip was guided by a negative-pressure airflow, and a shear knife coordinated with airflow was used to perform harvesting. Another accurate tea plant harvesting model was developed by Wang et al. (2021). Artificial neural network models were established using modeling parameters, such as shoot length, stem length, shoot angle, and growth time. The results showed good performance for predicting picking indicators. Motokura et al. (2020) implemented complex movements for manual picking using machine finger gripping, robotic arms, and motion control algorithms, thereby effectively harvesting tea shoots. Jia et al. (2022) designed a handheld tea leaf picker. By simulating human fingers to lift and pull tea leaves, the picker achieved a picking success rate of 74.3%. Miao (2019) designed a bionic picking finger by analyzing the mechanics of tea shoot tips. The optimization and analysis of the picking structure realized using genetic algorithm and MATLAB resulted in a 90% success rate for finger picking. Current tea shoot pickers are still based on point-to-point sequential picking in a three-dimensional (3D) space. However, such methods are inefficient and can cause damage to the picked tea shoots, thereby posing difficulties for application to actual tea gardens.

Machine vision technology has been extensively applied in the study of tea shoot tip identification. Yan et al. (2022) proposed an MR3P-TS tea shoot tip identification and localization model. This effectively identified overlapping tea shoots in tea plantations. Chen and Chen (2020) trained a region-based convolutional neural network (R-CNN), referred to as Faster R-CNN, to detect one-bud–two-leaf regions in images. A fully convolutional network (FCN) was then trained to identify the picking points in these regions. The experiments showed that the R-CNN model achieved an accuracy of 79% and a recall of 90%. Meanwhile, the average accuracy of the FCN was 84.91%. Yang et al. (2021) developed a procedure for tea shoot identification using the You Only Look Once version 3 (YOLO-V3) model. The intersection of the bottom edge of the marking frame and shoot was used as the selection point. The shoots were removed using a robotic arm. Zhu et al. (2022) used Faster R-CNN to construct a model for tea shoot detection in complex backgrounds. This algorithm outperformed the conventional algorithm in terms of the root mean square error. Li et al. (2021) developed a tea shoot identification model using the YOLO model. Subsequently, a red–green–blue (RGB)-depth camera was used to obtain point cloud data and RGB images; the shoots were finally picked off by a robotic arm, achieving a harvesting success rate of 83.18%. Xu W. et al. (2022) combined YOLO-V3 and DenseNet201 to accurately detect tea buds; the accuracy of tea bud detection was 95.71%. Zhang et al. (2021) used the Shi-Tomasi algorithm to identify tea shoots and Otsu algorithm to segment them. Finally, the picking point information was obtained using the skeleton algorithm and corner point detection. The picking success rate was 85.12%. Li et al. (2022) used the YOLO-V3-spatial pyramid pooling algorithm to achieve fast tea bud detection, achieving a detection speed of 15.9 fps. Yang et al. (2019) implemented an improved YOLO-V3 deep CNN algorithm for shoot picking point recognition; the accuracy of the training model was 90%. Qian et al. (2020) proposed a tea bud segmentation method (referred to as “tea-sprout segmentation network”) based on an improved deep convolutional codec network. The experimental results demonstrated the good segmentation performance. Peidi et al. (2018) used an improved k-means algorithm to identify tea leaves. The experiments showed that the algorithm improved young leaf segmentation in tea images. These aforementioned algorithms are based on the identification of tea shoots and localization of picking points in 3D space, which require extremely high accuracy of the picking actuator. However, its high cost poses difficulty for practical production. Moreover, the accuracy of recognition and localization algorithms deteriorate in actual environments. As such, none of these models can accurately calculate the location of the picking point. In addition, the low computing speed of recognition and localization algorithms limit the picking efficiency improvement.

The aforementioned scholars have performed in-depth studies of tea shoot identification and harvesting; however, the following problems remain in practical applications: 1) current research on tea identification is aimed at 3D imaging spaces, and the methods struggle to accurately locate the picking point when picking in the field. 2) Current tea pickers damage tea shoots during picking and have low picking efficiencies. 3) The high cost of hardware and picking execution components required to implement the current tea identification algorithms restrict their practical applications. Hence, to achieve continuous, accurate, and efficient harvesting of established tea shoots, this paper proposes a two-dimensional (2D) perspective-based selection scheme of tea shoot picking point location for standardized tea plantations. First, a semiclosed picking system was designed to organize tea stalk into strips. Subsequently, a camera was used to photograph tea leaves from the side, and a You Only Look Once version 5 (YOLO-V5) model, skeleton algorithm, and curve growth were combined to obtain the extract the coordinates of the picking point. The final combination of an S-curve damping algorithm and path-planning control picker facilitated the fast and low-loss picking of tea shoots.

## 2 Materials and methods

### 2.1 Growth parameters of tea shoots

**Figure 1:**
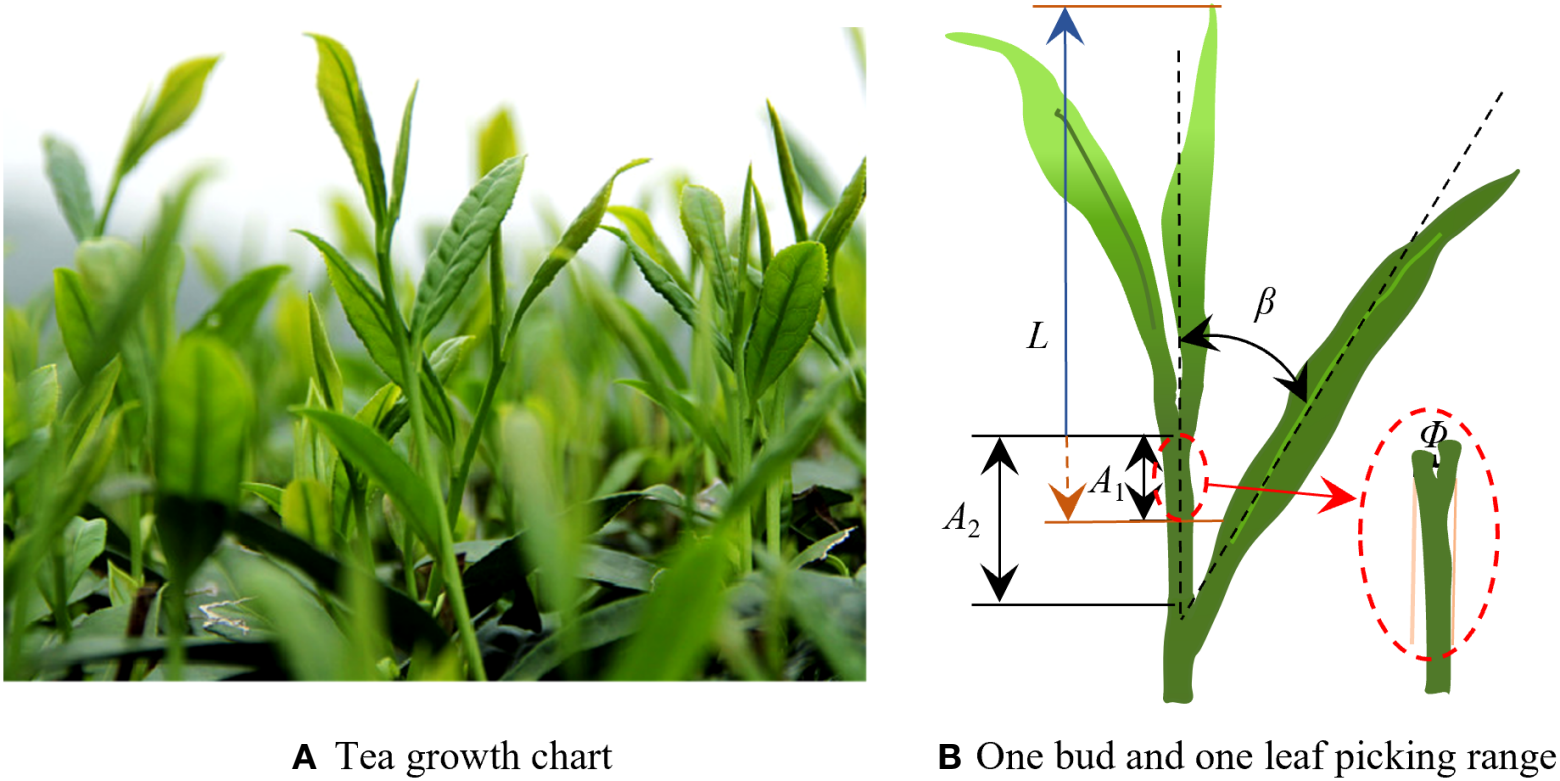
**(A)** Growth state of tea shoots and **(B)** tea shoot picking diagram.

The research objects of this study are the established tea shoots in a standardized tea garden. The tea leaf growth conditions during the harvesting period are shown in **Figure 1A**. When designing a picker suitable for standard tea plantations, the tea shoot growth parameters were first determined. The growth heights and locations of the tea shoots on the surface of the tea canopy determine whether they can be picked intact. In this study, a tea tree was randomly selected from a standardized cultivated tea plantation. The interval between the tea shoot tips on the tea canopy surface was measured to be 50 mm–80 mm, the tea leaf length was 700 mm, and the growth height of the tea shoot tip measured from the tea canopy surface was 50 mm–110 mm. The number of tea shoots per unit area accounted for 15%–20% of the picking operations (Zhu, 2022). The growth parameters related to the tea shoot tips are shown in **Figure 1B**, and the specific parameters are listed in **Table 1**, where *L* is the length of the tea shoot, *Φ* is the diameter of the stalk at picking, *A_2_* is the pickable region, *β* is the leaf angle, and *A_1_* is the optimal picking region.

**Table 1:** Physical parameters of the tea shoot tips.

Material type	*L*/mm	*Φ*/mm	*A_2_*/mm	*β*/°
One bud one leaf	27.5–30.2	1.1–2.3	11.2–15.3	45.6–54.2

### 2.2 Design of the continuous tea shoot picking system

**Figure 2:**
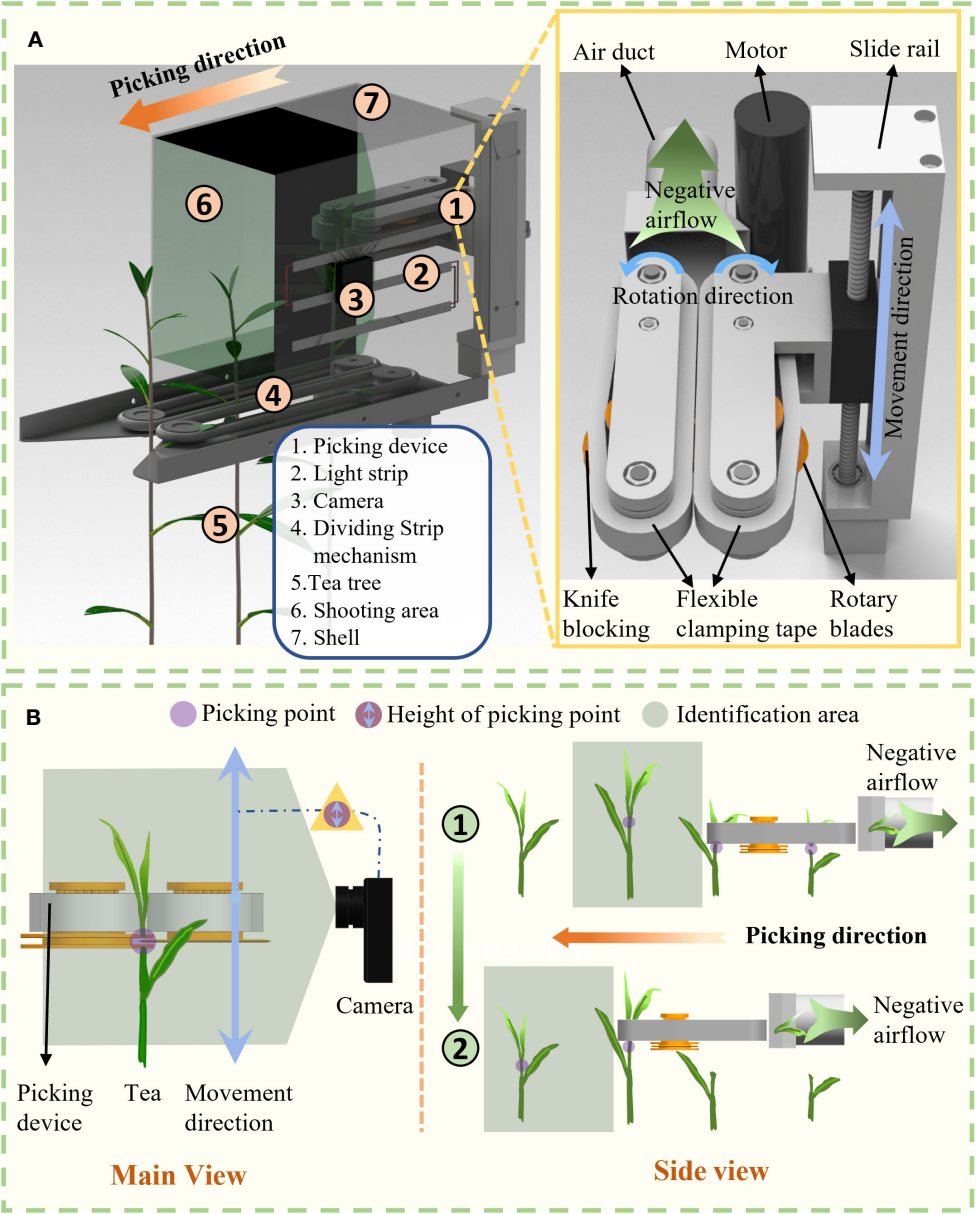
Picking system **(A)** configuration and components and **(B)** operation diagram.

The structure of the tea shoot picking system is shown in **Figure 2A**. The picking system consists of seven units: aligning unit, guiding unit, identification camera, light source, housing, vertical track, and picker. The picker comprises two flexible conveyor belts located opposite each other to avoid damaging the tea shoots during clamping. A circular blade and symmetrical blocking blade are set in the middle of the conveyor belt. The tea shoots are harvested and transported to the back of the device *via* a negative-pressure air tube. The picker is mounted on a vertically moving track mounted on top of the housing. The light strip and camera are fixed on the same side of the track. Three light strips are evenly distributed to illuminate the tea leaves in front of them, and the camera is mounted between the light strips. The aligning mechanism is installed at the front bottom of the housing. The camera and light strip operate in the space between the front of the aligning mechanism and front of the picker. The shooting range of the camera extends from the upper part of the aligning mechanism to the top of the housing. The individual components in the figure are as follows: 1: picking mechanism; 2: light belt; 3: camera; 4: guiding mechanism; 5: tea leaves; 6: bar-clamping mechanism; 7: identification area; and 8: housing.

The operation diagram of the picking system is shown in **Figure 2B**. The purple dots in the figure denote the tea shoot picking points determined by the recognition system, the purple circles and arrows show the picking point information transmitted by the recognition system, and the gray area shows the recognition area of the camera. The figure illustrates the working process of the picking system in both the main and side views. In the main view, the tea leaves are clamped by the aligning mechanism before they enter the shooting area of the camera, where they are positioned vertically in a strip. The camera captures a picture of the tea leaves and uses this as the input to the recognition algorithm. The algorithm determines the location of the tea shoots and sends the picking point information to the control system. The control system controls the track to move the picker up and down to reach the correct height for shoot picking. In the side view, the entire picking mechanism moves at a constant speed in the picking direction under a negative-pressure airflow, which continuously sucks the tea shoots. In the picture, the first process picker reaches the picking point of the tea leaves and is about to pick them. Meanwhile, the young tea leaves at the back are sucked by the negative-pressure airflow. Once the shoots are picked, the picker performs a second process. The picker moves upward. Before the front end of the picker reaches the tea shoots, the picker has already reached the corresponding height and is on standby for the next cycle. The cycle is repeated until the picking operation is complete.

### 2.3 Picker workflow and control algorithms

As tea leaves grow in a disorderly manner, the picker collides with the leaves as it moves from the current picking position to the next one. Moreover, the acceleration of the picker during motion can produce vibrations, leading to misalignment of the picking point. To prevent the conveyor clamping mechanism at the front of the picker from accidentally touching the tea leaves, its picking path should be planned according to the growth characteristics of the tea leaves. Furthermore, the movement speed of the picker must be planned to reduce the impact of vibrations, which can reduce the accuracy and damage the tea shoots.

**Figure 3:**
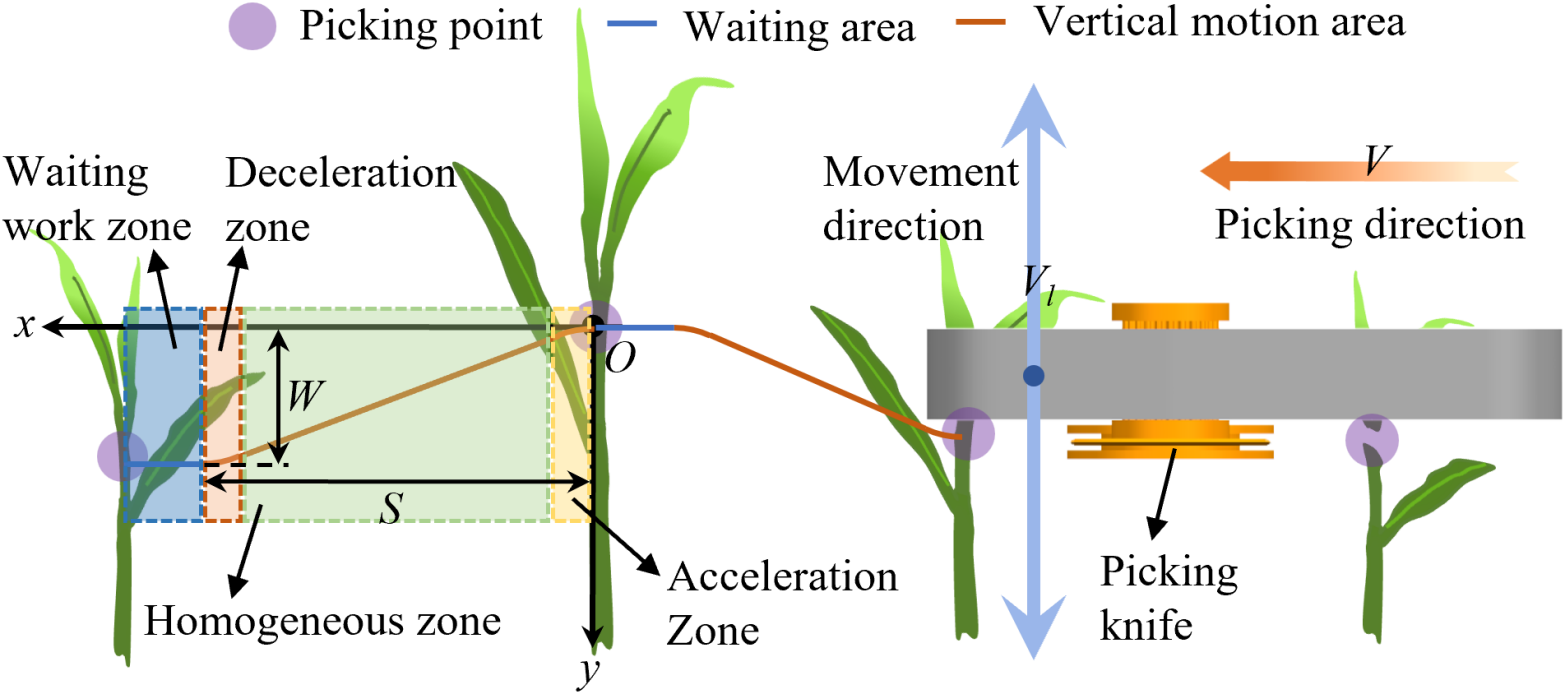
Picker speed control diagram.

In this study, the picker’s path was planned, as shown in **Figure 3**. The dashed blue, orange, green, and yellow boxes in the figure denote the waiting area, picker-deceleration area, area where the picker moves vertically and uniformly to the next picking point, and picker-acceleration area, respectively. The picker can move to the left at a constant speed *via* the picking mechanism. Meanwhile, the picking knife can move up and down. As the picker is always in uniform motion, the actual path of the picker forms an S-shaped curve when moving from the current position to the next. When the picker moves to the next position after picking from point O, it must accelerate to a set value in the vertical direction. Thereafter, it continues moving at the same speed. When the set position is about to be reached, the picker decelerates. When the speed of the picker in the vertical direction becomes zero, the picker reaches a predetermined position and retains the same vertical height while waiting for the tea shoots.

The tea leaf distribution interval was measured to be 50 mm–80 mm in the previous section. A tea protection zone, which is the waiting area denoted by the dashed blue box in **Figure 3**, was established according to the tea leaf growth characteristics to prevent the picker from accidentally pinching the tea leaves. The width of the waiting zone was 10 mm. The orange, light yellow, and light red curves represent the picking trajectory, acceleration zone, and deceleration zone, respectively.

We chose S-type acceleration and deceleration functions to plan the acceleration and deceleration of the trajectory and reduce the movement of the picker, which produce mechanism vibrations that affect the picking accuracy. When the picker moves horizontally, the horizontal speed *V* is constant. When the picker moves from point O to the next target picking point, the speed of the picker in the vertical direction must quickly reach the maximum set value, such that the picker can quickly move down to avoid the tea leaves. When the picker is about to reach the height of the tea picking point, it should decelerate vertically. The velocity control function is established in the trajectory of the acceleration and deceleration sections of the curve, given as follows:


(1) $$V_{l}\left(s\right)=v_{l0}+\left(v_{lmax}-v_{l0}\right)\frac{1}{1+e^{-as+b}}\;\left(0<s<S_{l}\right)$$

where *v_l0_* is the initial velocity (m/s), *v_lmax_* is the maximum velocity (m/s), *V_l_* is the velocity at different displacements (m/s), *a* is the curve coefficient, and *b* is a constant.

**Figure 4:**
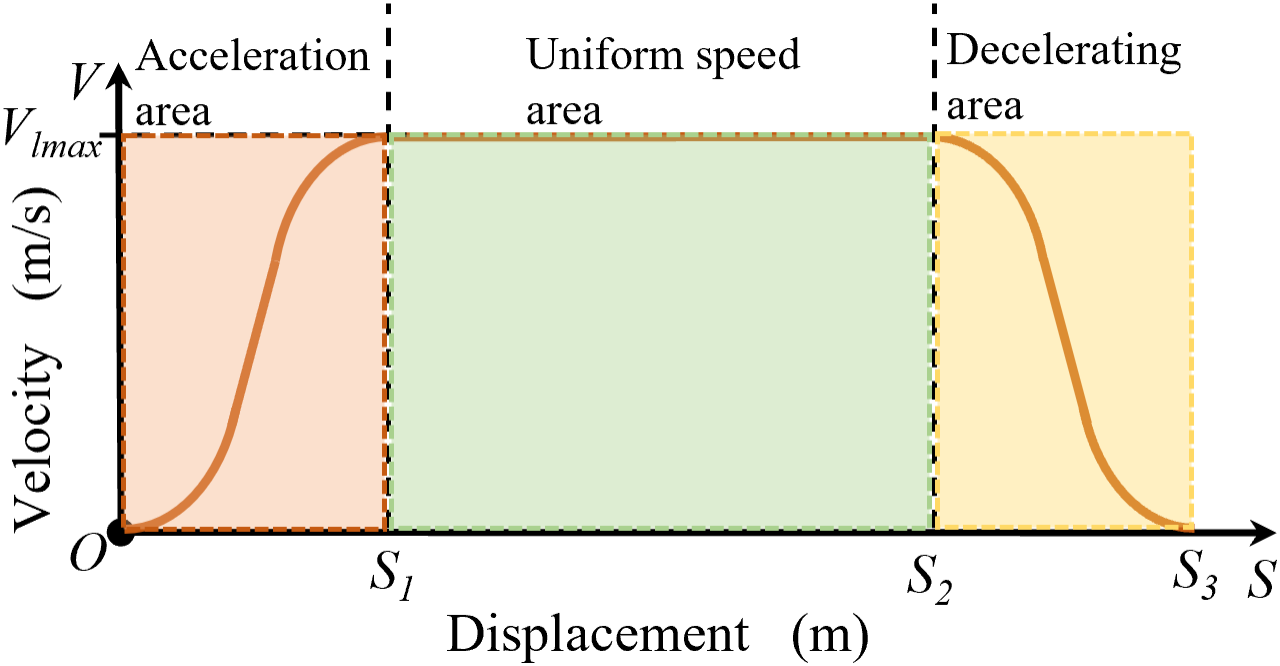
Vertical velocity planning diagram.

As the S-curve function has a long and slow initial growth interval, a constant term *b* was added to shift the function curve by a specific distance, thereby offsetting the effect of the slow function value growth. The movement of the picker from the current position to the next forms a complete picking cycle. As shown in **Figure 4**, the picker is controlled using acceleration and deceleration functions at the front and end of the motion trajectory because it must quickly reach the maximum speed. The velocity changes at the front and back ends exhibit the same trends. The speed in the acceleration section increases slowly and then rapidly before slowly decreasing. Meanwhile, the speed in the deceleration section decreases slowly then rapidly before slowly decreasing to zero.

Owing to uncertainty in the movement distance of the picker lead hammer, this study set the acceleration and deceleration zones at a fixed distance *S_1_*, where the uniform zone displacement *S_y_* is expressed as follows:


(2) $$S_{y}=S-2S_{1}$$

An excessively large *S_1_* will cause erroneous clipping of adjacent tea leaves, whereas an excessively small *S_1_* will cause failure in the tea picking. Thus, *S_1_* is set as the distance from the front end of the picker clamping to the front end of the picking knife to ensure that the picker can accurately pick the tea leaves. The initial velocity is zero. When the distance is *S_1_*, the velocity is increased to the maximum value *v_lmax_* to obtain the velocity control function using the equation:


(3) $$V_{l}\left(S\right)=\frac{v_{lmax}}{1+e^{-a\left(S-b\right)}}\;\left(0<s<S_{1}\right)$$

Here, the maximum speed is 0.12 m/s, *S_1_* is 0.04 m, *a* is 0.0067, and *b* is 0.02.

### 2.4 Identification model and picking point location

The field environment of tea plantations has complex lighting systems and low contrast between young and old tea leaves. Therefore, previous image processing and deep learning approaches based on 3D perspectives suffer from limitations in tea shoot tip recognition. In this study, we compared previous methods to establish a tea shoot tip identification model and extract picking point information through a 2D imaging perspective using YOLO-V5 combined with a skeleton algorithm (Zhang L. et al., 2022; Zhang P. et al., 2022; Huang et al., 2023; Li et al., 2023).

#### 2.4.1 Data acquisition

The data used in this study were obtained by taking photographs at the Agricultural Park of Anhui Agricultural University in Hefei, Anhui, China. The shooting time was from mid-March to early April 2022. The row spacing was 0.5 m, and the tea variety was Shucha early. The image acquisition device was an MS-748 camera (specifications: technology: complementary metal oxide semiconductor; pixel size: 3 μm × 3 μm; frame rate: 30 fps; shooting angle: 140°). The distance between the camera and tea leaves also affects the accuracy of tea recognition. Therefore, in this study, the images of the tea leaves were collected at a distance of 5 cm–20 cm. As light influences the photography results, the tea images were collected in the morning, midday, and evening. A total of 971 tea leaf images were collected.

#### 2.4.2 Dataset processing

This study used a labeling software to manually label 971 collected tea leaf images. We expanded the number of images *via* color adjustment and mirror flipping to improve the diversity of the dataset and allow it to adapt to more complex environments. In particular, 971 datasets were amplified to obtain 5,616 images as the final dataset. The training, validation, and test sets comprised 70%, 20%, and 10% of the dataset, respectively.

#### 2.4.3 Identification model

**Figure 5:**
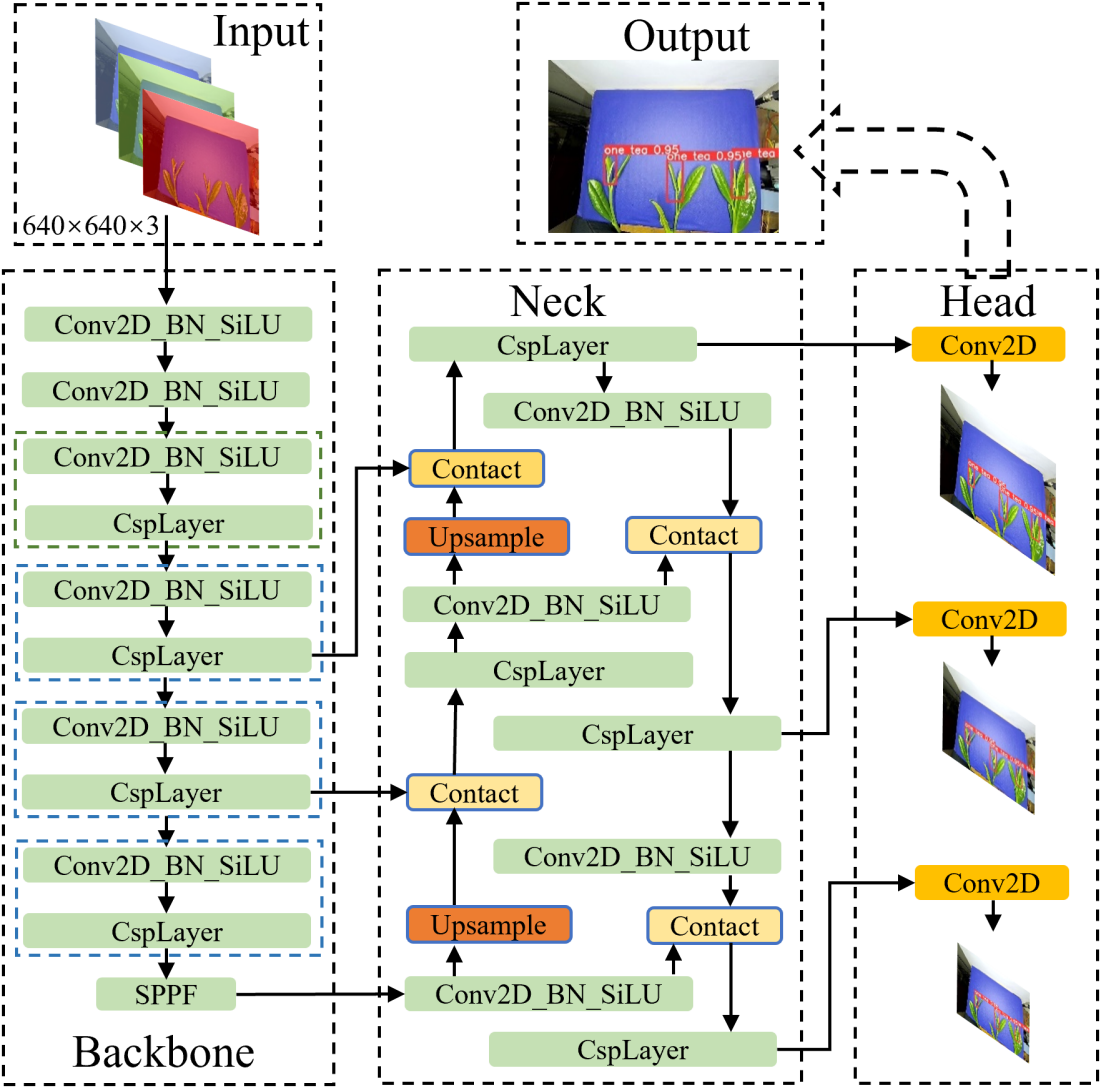
YOLO model processing flowchart. SPPF, Spatial Pyramid Pooling - Fast; CspLayer, Cross Stage Partial; Conv2D-BN-SiLU, Convolution 2D+ Batch Normalization + Activation; conv2D, Convolution 2D.

The network structure of the tea shoot tip identification model is shown in **Figure 5**. The YOLO-V5 target detection model is an end-to-end model offering faster recognition. The background behind the tea plants was integrated into the recognition environment to facilitate fast recognition in the YOLO-V5 model, thereby further improving the recognition accuracy.

#### 2.4.4 Test platform and evaluation index

The computer configuration used for model training was a CPU processor (12th Gen Intel^®^ Core™ i7-12700H 2.70 GHz) with a graphics card NVIDIA RTX3060 GPU and 16 GB of RAM. The model ran on a Windows 11 Professional 64-bit operating system. The software tool was PyCharm 2021.3.2, and the experiments were implemented in the Pytorch framework. The initial learning rate of the weights was 0.001, and the decay coefficient was 0.0005. The total number of model training iterations was 300.

We used recognition accuracy *P_C_*, recall *R_C_*, and mean average precision (MAP, *F_C_*) as evaluation indexes to evaluate the effectiveness of the trained recognition model for recognizing tea shoot tips. The formulas for each index are as follows:


(4) $$P_{C}=\frac{TP}{TP+FP},$$

(5) $$R_{C}=\frac{TP}{TP+FN},$$

(6) $$F_{C}=\frac{2P_{C}R_{C}}{P_{C}+R_{C}},$$

where *TP* is the number of correctly identified tea shoots, *FP* is the number of incorrectly identified tea shoots, and *FN* is the number of unidentified tea shoots. The time between when the tea leaves enter the camera’s field of view and the recognition model obtains the location information of the tea shoot tips also reflects the performance of the model. In particular, the shorter the average processing time is, the better the model.

### 2.5 Picking point location algorithm

**Figure 6:**
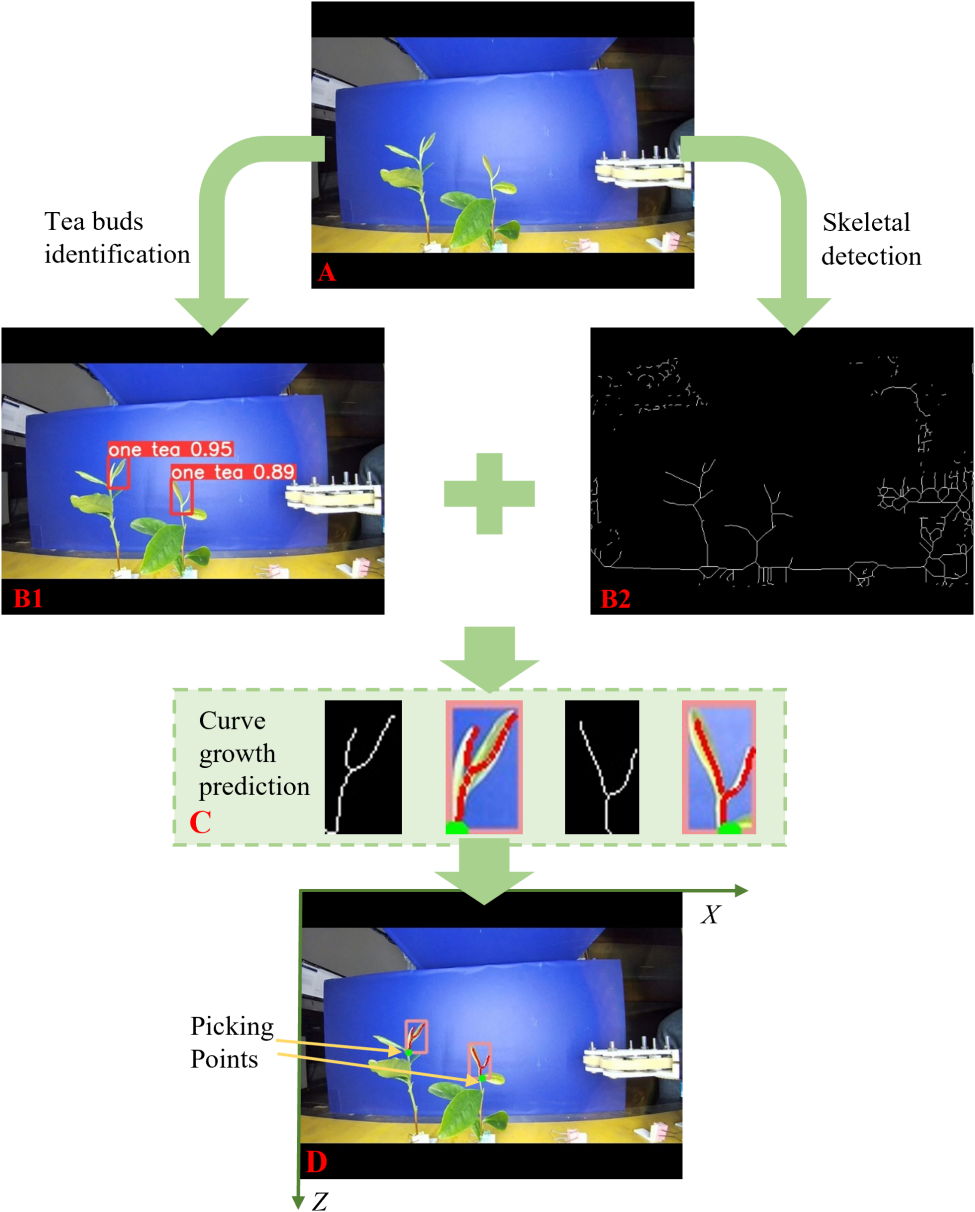
Tea tip recognition and picking point positioning flowchart. **(A)**: pictures taken of tea leaves; **(B1)**: framed picture of tea leaf shoots; **(B2)**: extracted skeleton information picture; **(C)**: drawing growth curve pictures; **(D)**: determine the picking point picture.

The tea shoot tip recognition model obtains the position of the tea shoot tip in the picture; however, it cannot control the picking mechanism to reach the exact position to pick it. Therefore, the exact picking points of the tea shoots were determined. The top of the tea shoot was crossed by two leaves, its bottom formed a separate stalk, and the area above the leaves was blank. This study proposed a skeleton algorithm and curve growth method to determine the exact location of the picking point of the tea shoot tip. Only the X- and Z-values of the actual coordinate system of the tea shoot tips were required for the identification. The analysis flow of the picking point location of the tea shoot tip is shown in **Figure 6**.

1. As shown in **Figure 6A**, we obtained a picture of the tea leaves (**Figure 6A**), which was fed into the recognition model. After the YOLO-V5 model identifies the tea shoots, it uses the bounding box to frame the shoots out. The algorithm obtained the coordinate information of the four corner points of the marker box. When a tea shoot was selected, its upper right corner was marked with the type and probability of this object as shown in **Figure 6B1**.

2. As the picking point position was fixed at 4 mm from the end of the tea shoot, the location of the intersection of the shoot buds and leaves was first used to determine the exact location of the picking point, which is the end of the shoot tip. As shown in **Figure 6B2**, we used the skeleton algorithm to create a skeleton image of the objects within the image. The skeleton image was represented using a white line with a width of one pixel. In this study, we used the fast parallel algorithm by Zhang and Suen (1984) to extract the skeleton information of the tea shoot tips (Zhang and Suen, 1984; Layana Castro et al., 2020). First, the collected images of the tea shoot tips were binarized, and a refinement method based on the mathematical morphology was applied to refine the juxtaposed pixels of the leaves and stalks in the tea shoot images.

3. The picking point analysis model obtained the coordinate information of the four corner points of the tea shoot marker box from the recognition model to obtain accurate coordinate information on the tea shoot picking point. As shown in **Figure 6C**, the image of the bounding box for the tea shoot tip and corresponding image of the tea shoot tip skeleton were simultaneously extracted. The number of pixels in them was scanned horizontally per line. Scanning was stopped when multiple pixels were present in a single line. The growth curve function of the stem was established using the position of the intersection point as the starting point and downward along the skeleton curve in the direction of the stem growth to accurately determine the distance between the picking point and tea shoot tip. Thus, the actual growth curve function of tea stalks based on the actual coordinate system scale was determined.

4. As shown in **Figure 6D**, the vertical coordinate along the function curve was reduced by 4 mm and set as the location of the picking point depending on the determined function. This point was marked in the graph with a green dot. Thereafter, the green dot was mapped for the complete image to yield the coordinate information of the tea shoot picking point. The coordinate information in the image was calibrated to obtain the actual coordinate information for the tea shoot picking point.

### 2.6 Tea tender tip identification and picking test bench

#### 2.6.1 Tender tip identification and test bed design

**Figure 7:**
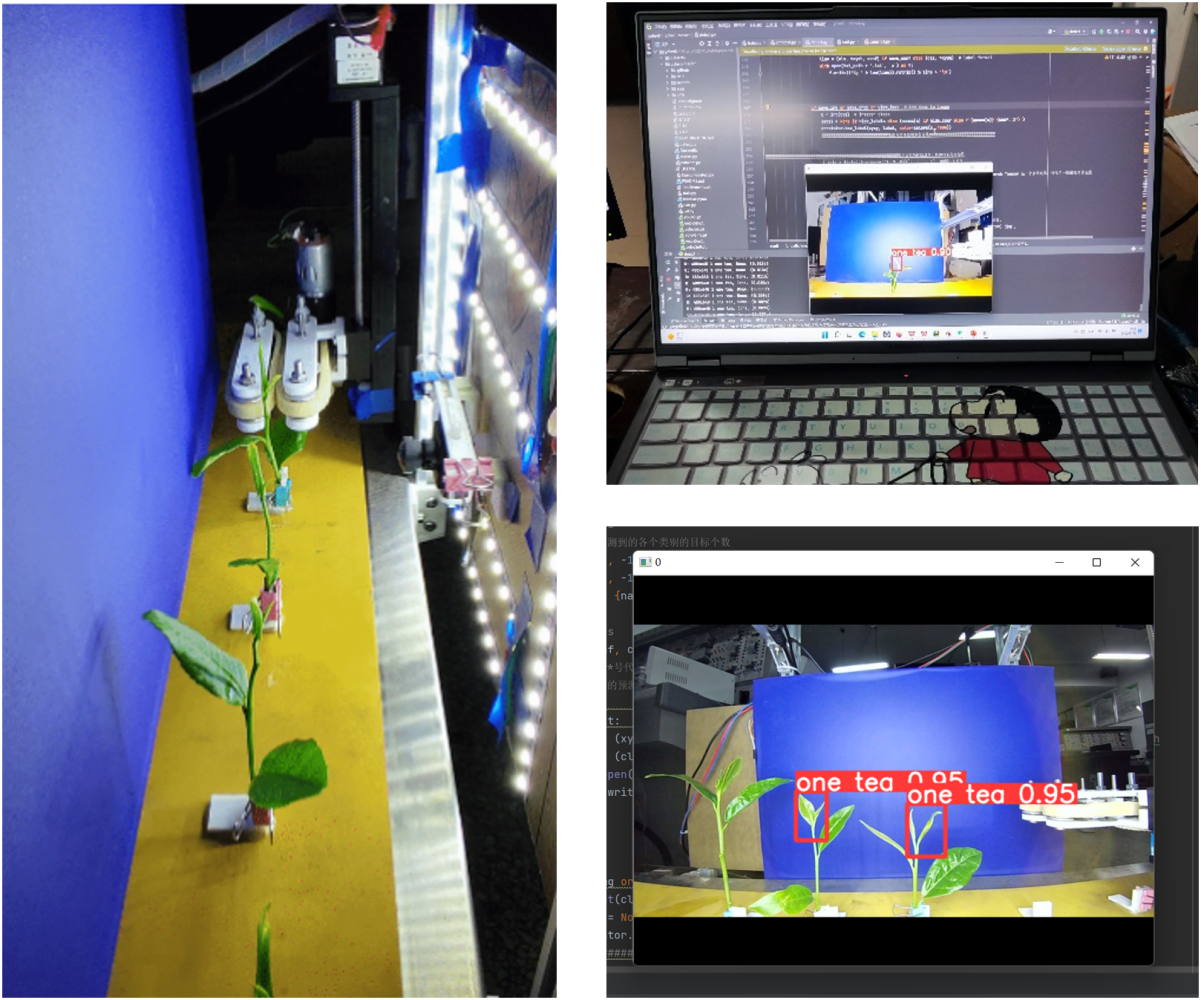
Test bench structure.

A tea shoot identification and picking test bench, as shown in **Figure 7**, was designed to verify the accuracy of the identification and picking point location algorithm in an actual environment, the picking mechanism design, and the stability of the control system. The main structure consists of a conveying device, picking mechanism, control system, and identification system. The conveying device is a conveyor belt mechanism modified to fix tea stalks on it using an L-shaped fixing frame. The picking mechanism is a continuous picker designed in Section 2.2 Design of the continuous tea shoot picking system. The control system primarily consists of the respective controllers of the conveyor belt, stepper motor, DC motor, and lower computer that receives the upper model number.

First, the tea leaves with stalks were fixed on the conveyor belt according to their actual growing conditions. Next, the recognition system was initialized. We set the conveyor belt to rotate forward at a constant speed. The tea leaves entered the identification area at a uniform speed, and the camera captured pictures of the tea leaves to obtain the picking point information of their tender tips. Next, the algorithm generated the speed control function to move the picking mechanism, and the lower computer instructed the mechanism to move to the calculated height. The tea leaves then entered the picker to pick the shoot tips off. The stalks were continuously transported backward, and the tea shoots were transported to the rear collection area. The trial was completed when all of the tea shoots within a row have been picked.

#### 2.6.2 Image and coordinate system calibration

**Figure 8:**
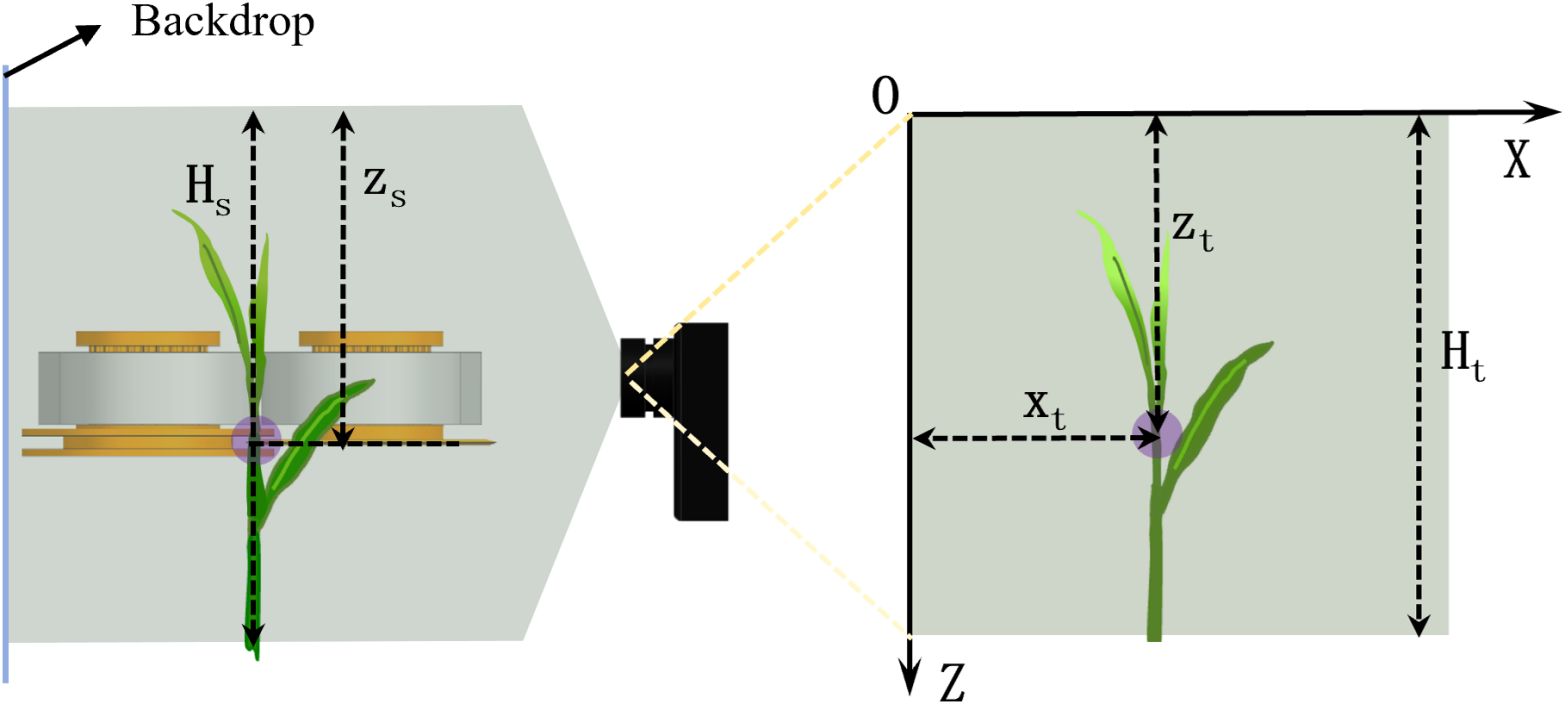
Camera calibration diagram.


As the coordinates in the image are determined in terms of pixel points, the coordinates of the picking points in the image should be calibrated to the actual coordinate system used to control the picking mechanism. A calibration ruler was placed at a fixed position on the tea leaves during actual calibration. The ruler was fixed parallel to the plane upon which the tea leaves were placed and oriented vertically. As shown in **Figure 8**, the recognition model determined the picking point location of the tea shoot using the upper left corner of the image as the origin. The Z-axis was on the left, and the X-axis was at the top. The number of pixels in the image was set as the coordinate value. *H_t_* in the image denotes the height of the picture, and *Z_t_* is the height of the picking point. In the actual coordinate system, *Z_s_* is the height of the picking point and *H_s_* is the height of the shooting area. Their relationship is expressed as follows:

(7) $$\frac{Z_{t}}{H_{t}}=\frac{Z_{s}}{H_{s}}$$

*X_t_* in the figure indicates the position of the picking point in the image in the transverse direction, and its value in the actual coordinate system can be set at the same scale.

**Figure 9:**
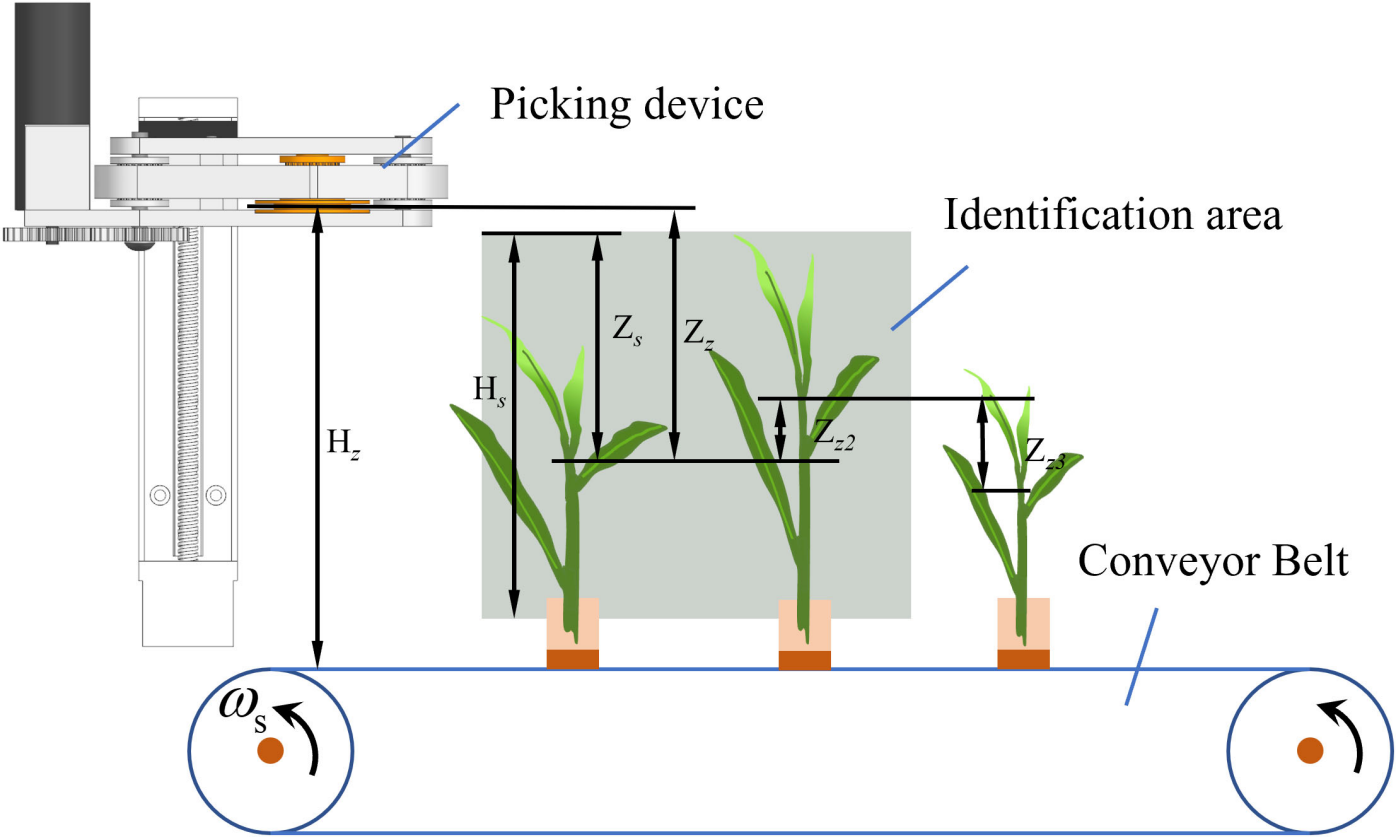
Picking mechanism and picking point position diagram.

The locations of the picking mechanism and picking point are schematically shown in **Figure 9**. When the picking mechanism was at its highest position, the height at which the picking blade was located was taken as the starting point to determine the height of the blade position with respect to the picking point. Once the blade reached the current picking point, this position is used as the starting point to calculate the height distance to the next position. According to actual measurements, *H_z_* = 212 mm and *H_s_* = 196 mm.

## 3 Results and discussion

### 3.1 Model validation

**Figure 10:**
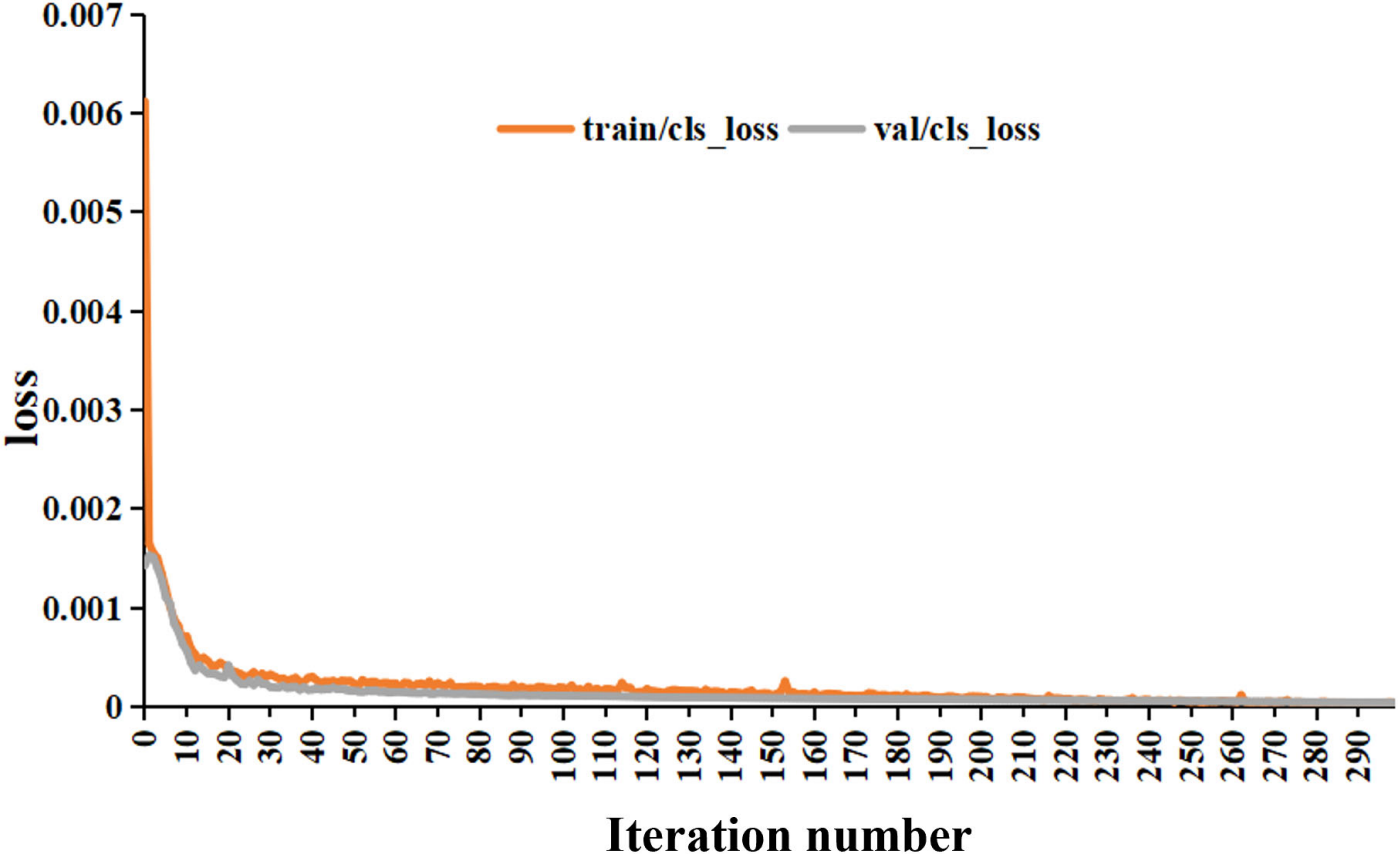
Model training results.

**Figure 11:**
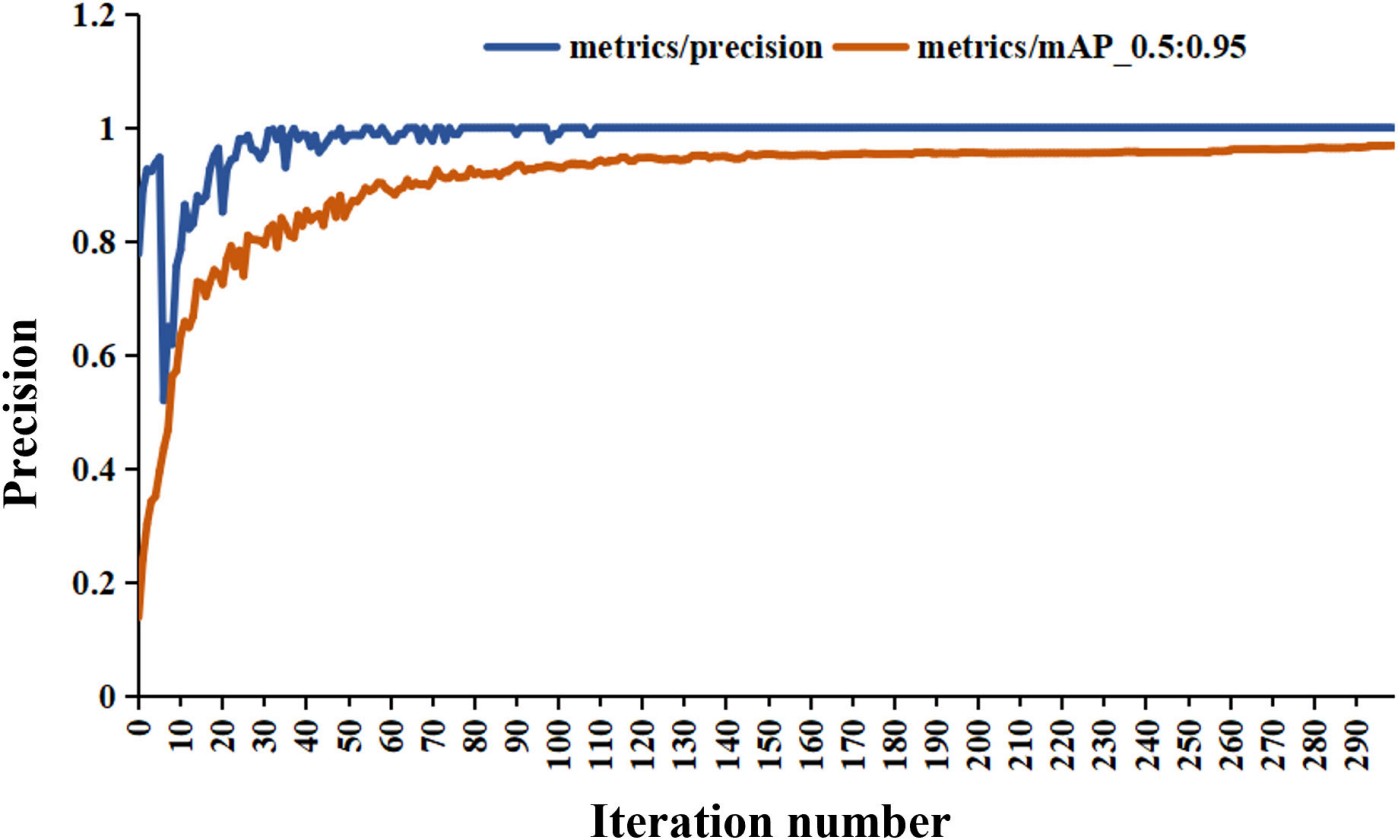
Model training accuracy.

The tea pictures were imported into the YOLO-V5 training model, and the evaluation index results were obtained, as listed in **Table 2**. The results for the training and validation sets of the model are shown in **Figures 10**, **11**, respectively. After 300 iterations, the recognition accuracy of the validation set reached 99.9%. The loss values for the training and validation sets were 3.5e–5 and 4.33e–5, respectively. The MAP value (0.5:0.95) after 300 iterations was 0.97.

**Table 2:** Model training parameters.

*F*c	*P*c	*R*c	MAP (0.5)	MAP (0.5:0.95)
0.789	0.83	0.75	0.99	0.96

### 3.2 Effect of speed on the recognition accuracy

We investigated the accuracy of the recognition system at different speeds to test its performance. We used a speed meter (DT22360; Guangzhou Rongmei Electronic Co., Ltd.) to measure the linear speed of the conveyor belt surface as the speed of tea movement. A group of tea leaves was selected as the test object, and different tea leaf moving speeds were achieved by varying the speed of the conveyor belt. The recognition accuracy was obtained by testing; the results are shown in **Table 3**. When the speed of the conveyor belt exceeded 0.47 m/s, the accuracy of the tea shoot identification system started to deteriorate. The accuracy rate decreased faster as the speed was increased. When the speed was lower than 0.47 m/s, the accuracy of the tea shoot identification system stabilized at 100%.

**Table 3:** Identification accuracy at different speeds.

Tea movement speed/(m/s)	Recognition accuracy/%
0.20–0.47	100
0.60–0.87	80–75
1.00–1.13	61–57

**Figure 12:**
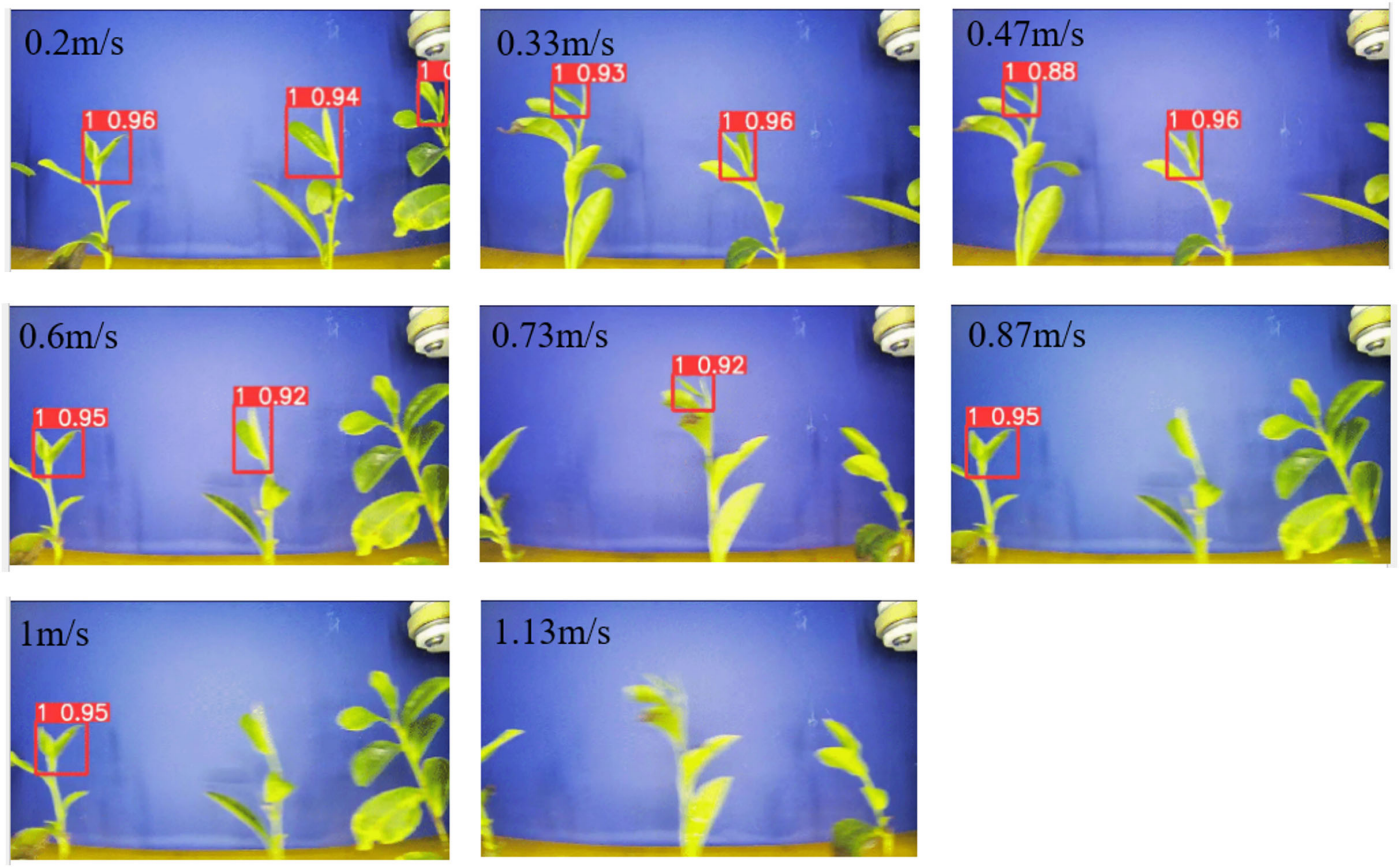
Recognition effect for tea shoots at different speeds.

The recognition effect of the tea shoot tips at different speeds is shown in **Figure 12**. The same group of tea leaves gradually became blurred as the speed was gradually increased. A blurred screen reduces the recognition accuracy. Nonetheless, the recognition system can recognize some of the tender tips, which demonstrate the high stability of the recognition system. When the speed increases, the accuracy of the tea shoot identification for the same tea shoot decreases to 0.96 at 0.2 m/s and 0.95 at 1 m/s. Furthermore, the processing speed of the recognition system exhibits a certain lag, suggesting that the image of the current frame was not successfully intercepted for recognition and analysis and some of the tea shoot is missing.

As tea leaves pass across the camera lens from left to right, several inconsistencies were observed between the tea leaf images from the left and right sides. Different camera angles can obscure the tea shoots. When the shoots are obscured, the recognition system misidentifies them, resulting in deviations in the picking point information. As the speed was gradually increased, the recognition system becomes less stable. At high speeds, some tea shoots were not identified even though the results for the identified shoots are correct. We narrowed the camera’s field of view to the central section of the image to ensure the stability of the system. The field-of-view width is 200 pixels. Moreover, the number of samples in the model was increased by making it a separate object to avoid large leaves from shading the shoot tips. Furthermore, the picker moved to the very top to avoid large leaves when the system recognizes them.

### 3.3 Tea shoot identification and picking efficiency

The time taken for the system to identify and locate the picking point and perform picking was used as an index of the picking efficiency. The time required for each phase is shown in **Table 4**. The picking time is the total time spent from identification to picking completion. The average time taken by the model to identify the shoots was 30.25 ms and that to determine the picking point was 37.375 ms, and the average time taken to complete picking was 0.768 s.

**Table 4:** Time taken by different stages.

Serial number	Identification time/ms	Positioning time/ms	Picking time/s
1	32	35	0.767
2	29	40	0.769
3	30	38	0.768
4	28	37	0.765
5	33	36	0.769
6	25	36	0.761
7	34	39	0.773
8	31	38	0.769
Average	30.25	37.375	0.768

The improved model takes less time to identify the shoot and can satisfy the requirements of real-time and rapid shoot identification in the field. Increasing the speed of model recognition can further improve the efficiency. However, the too short model recognition time repeatedly recognizes the same tea shoot. The picking mechanism is limited by the speed of the slide; hence, the recognition model cannot be set to operate too fast (i.e., excessive speed can cause the rear picking mechanism to pick out of sequence). We added a time delay in the algorithm loop to ensure the accuracy of the picking mechanism. The interval between the video streams’ interception by the model was set experimentally as 0.7 s. The shoot picking test bench shows that the actual picking point position was within a ±3 -mm margin from the position determined by the model; thus, this satisfies the demand for accurate tea shoot positioning.

### 3.4 Results of the tea tender tip picking

**Figure 13:**
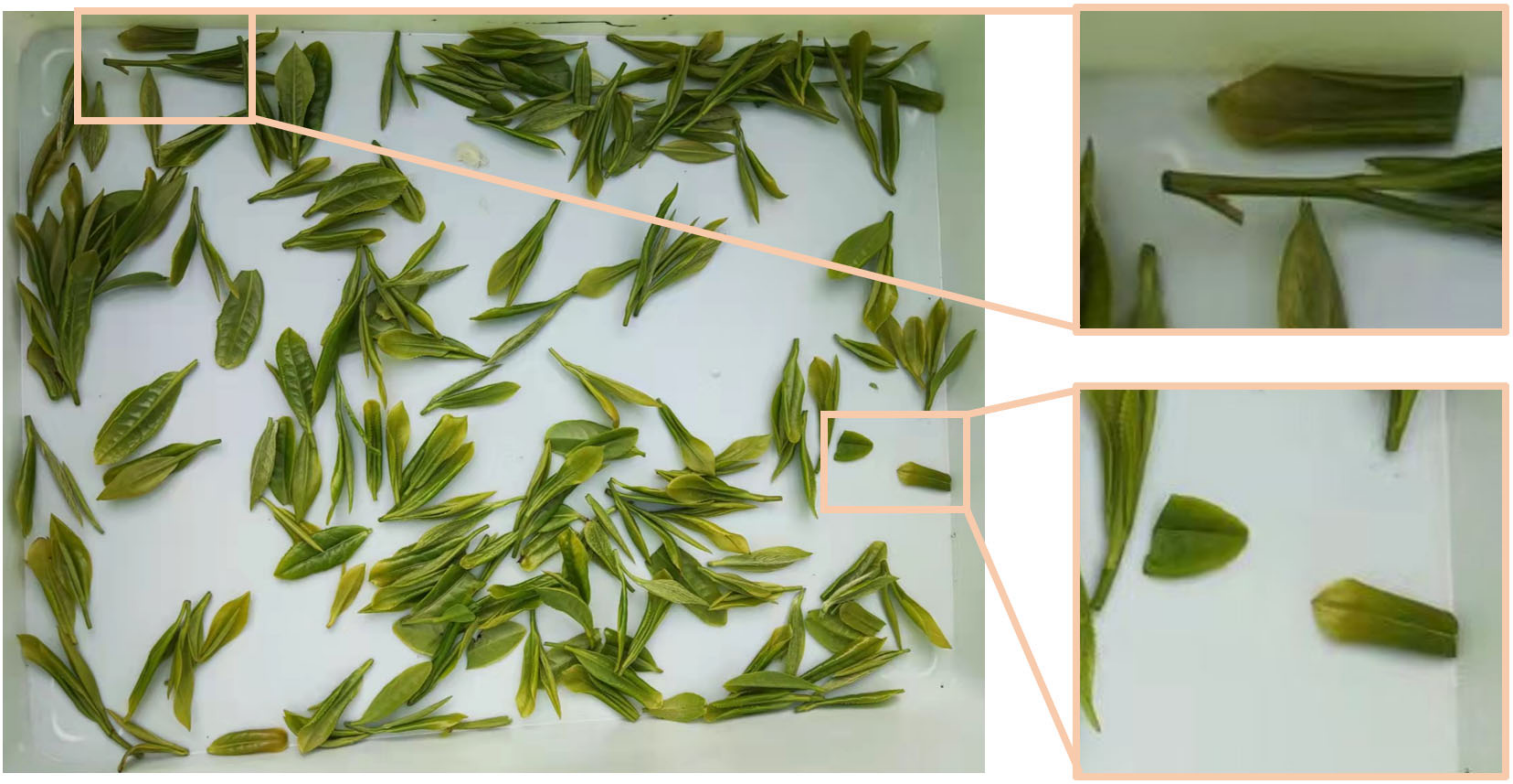
Tea shoot picking results.

We selected multiple picking locations along a single row in a tea garden to test the reliability of the tea shoot picking method designed in this study. The trial tested the identification, positioning accuracy, baring success rate, and picking success rate of the system. The success rate of strip clamping expresses the percentage of tea stalks successfully clamped and placed in an upright position as a proportion of the total number of tea stalks. Successfully picked tea leaves are those whose shoots are undamaged and intact and whose stalk lengths are less than 5 mm. The specific test results are shown in **Table 5**. The results of the picking test are shown in **Figure 13**.

**Table 5:** Tea shoot picking test results.

Number of test shoots	Number of successful strip clampings	Number of successful pickings	Success rate of strip clamping/%	Picking success rate/%
98	90	82	91.8	83.6

### 3.5 Discussion

As shown in **Figure 13**, most of the stalk lengths of the tea shoots meet the agronomic requirements. In addition, excessively long stalks can also be noted owing to the overgrowth of the tea leaves on an incline. The actual picking test showed that the actual picking point position was within ±3 mm of the model-determined position. The error is mainly attributed to the excessive bending of the tea shoot growth. In addition, the picking mechanism that shears the tea shoots can also cause deviations in the picking point. As such, the picking mechanism will be optimized from the principle of tea stalk mechanics at a later stage to further improve the picking accuracy.

During the system operation, the gripping component of the picker gathered larger leaves, which were shredded by the rear-picking knife. As such, broken leaves and shoots were transported to the collection box. Hence, the tea shoots should be screened after harvesting. However, repeated screening can further damage the tea shoots and reduce their quality. Therefore, the picking knife should be replaced in future research by a simulated manual lifting. Stem breakage but not leaf breakage was observed depending on the different breaking forces of the leaf and shoot stalks (Luo et al., 2022). A picker requires high control accuracy when picking. Moreover, identification should be separated from picking in the future to adapt to the picking requirements for a greater variety of tea leaves.

The coordinates of the end of the median line of the tea shoot tip were previously identified as the picking point location (Chen et al., 2022). This method resulted in the shredding of tea shoots during picking. In this study, identifying the picking point at 4 mm from the tail can avoid the shredding of the shoot tips being. Several scholars have set the location where the lower border of the marker box of the recognition model crosses the tea leaves as the picking point (Yang et al., 2019; Yang et al., 2021). In the 3D view, the lower border of the model was framed in an inaccurate position, causing the chopping of the tea shoots. By calculating the position of 2% of the total length of the tea shoot as the picking point, the average growth length of the tea leaves was obtained according to the growth characteristics of tea buds. Subsequently, the harvesting points were identified at a certain distance downstream (Li et al., 2021). As tea leaves grow in different states and different tea leaves will lead to different picking point locations, these methods cannot realize the precise picking of tea leaves.

Currently, several scholars are utilizing depth information to obtain the coordinates of tea shoot picking points in a 3D view. However, this cannot accurately localize the picking point, and the complex environment in the field can shift the localized picking point during picking. Meanwhile, some researchers have utilized robotic arms to pick tea shoots individually, which is not an efficient method. Previously, a tea shoot can be picked in 2 s–3 s (Yan et al., 2022). In this study, the picking of a tea shoot occurs in less than 1 s.

Several researchers adjusted the height of the overall cutter to coincide the cutter surface with the growth area of the tea shoots, thereby realizing efficient and precise harvesting. Nonetheless, precise selective picking is still not possible because the linear cutter picks a row of tea leaves at the same time (Tang et al., 2016). Thus, in this paper, standardized cultivation of tea leaves is proposed to further improve the picking efficiency and accuracy. Tea shoots are concentrated at the horizontal top of the tea tree. The camera laterally recognizes the tea shoots combed into rows and accurately locates the coordinates of the picking point. Meanwhile, the low-loss picker continuously picks the tea shoots. The streamlined picking program can provide new picking ideas for future tea shoot picking. However, standardized cultivation of tea leaves and optimal design of picking mechanism are still needed to achieve industrial application.

## 4 Prospect of tea shoot harvesting research

In this paper, a 2D side-view recognition picking scheme was used to improve the accuracy of 3D-view recognition localization. Continuous fast picking was realized by the continuous picking mechanism designed in this study. This provides new ideas and theoretical basis for continuous low-loss picking of tea shoots. However, there are still problems that need to be addressed for practical applications, as follows:

1. As a large amount of famous tea shoots need to be harvested in a short period of time, the identification harvesting methods in this paper satisfy the harvesting requirements; however, the current picking mechanism still suffers from low picking efficiency. This can be improved by adding robotic arms and picking components, which increases the picking cost. Further optimization of the picking mechanism is needed in the future to explore the options for continuous high-speed picking of tea shoots.

2. The picking mechanism still requires a knife cut for the final picking operation. The contact between the knife and tea stalks can have an oxidizing effect on the tea. As such, manual harvesting mechanisms can be further investigated in the future to realize tool-less harvesting that can achieve the desired quality.

## 5 Conclusion

In this study, a 2D imaging-based continuous picking system was designed to facilitate the continuous picking of tea shoots in standard tea plantations. The YOLO-V5 recognition model was combined with a skeleton algorithm and curve growth algorithm to achieve continuous recognition and localization of tea shoot tips. The proposed system facilitated the continuous picking of tea shoots. The following conclusions were drawn:

1. The 2D recognition perspective can improve the tea shoot recognition accuracy. The combination of the skeleton algorithm and curve growth increased the positioning accuracy of the picking points. After experimental validation, the maximum recognition speed of this method was 0.47 m/s. The identification accuracy, picking success rate, and picking efficiency for the tea shoots satisfy the requirements for practical applications.

2. The recognition accuracy of the validation set for the tea shoot tip recognition model was 99.9%. The average time required for picking was 0.768 s. The picker used an S-curve function to facilitate accurate control of the up and down movements. The shoot picking test bench test results showed that the actual picking point position was within ±2 mm of the modeled position. The picking success rate was 83.6%, which justifies the rationale and demonstrates the efficacy of the continuous low-loss harvesting.

3. This paper addressed the operation in standardized cultivated tea gardens. A continuous picking mechanism combined with a low-cost camera can decrease the cost of the picking system. Moreover, simplifying the identification and picking process reduced the time needed to pick a single shoot to less than 1 s. Moreover, this further improved the stability and accuracy of tea shoot identification.

We anticipate that the findings of this work will help improve the efficiency of automated tea shoot picking systems, thereby securing economic benefits for the tea industry.

## Data availability statement

The original contributions presented in the study are included in the article/supplementary material. Further inquiries can be directed to the corresponding author.

## Author contributions

KL: conceptualization, methodology, software. CC and ZW: supervision. KL: grammar review. KL and XZ: data curation, writing, reviewing, and editing. KL, WL, LC, and WC wrote the original draft. XZ, KQ, and CW: visualization, investigation. All authors contributed to the article and approved the submitted version.
